# Preparation Technique Affects Recipient Immune Targeting of Autologous Mesenchymal Stem Cells

**DOI:** 10.3389/fvets.2021.724041

**Published:** 2021-09-14

**Authors:** Aileen L. Rowland, Madison E. Burns, Gwendolyn J. Levine, Ashlee E. Watts

**Affiliations:** ^1^Department of Large Animal Clinical Sciences, Texas A&M University, College Station, TX, United States; ^2^Department of Veterinary Pathobiology, Texas A&M University, College Station, TX, United States

**Keywords:** mesenchymal stem cell, fetal bovine serum, bone marrow supernatant, horse, intra-articular, immunogenicity, equine, mesenchymal stromal cell

## Abstract

Fetal bovine serum (FBS) is used for MSC preparation in pre-clinical animal models and veterinary applications, recently in US clinical trials, and for MSC products with current foreign market authorizations. The effect of anti-bovine titers, which are common in animals and humans, has not been investigated. In the equine model, where anti-bovine titers are universally high due to routine vaccination, we evaluated the recipient immune response to autologous MSCs prepared with and without FBS. Preparation of MSCs with FBS resulted in post injection inflammation and antibody mediated cytotoxicity of MSCs when compared to MSCs prepared without FBS. Importantly, synovial MSC concentrations were reduced and LPS induced pain was higher, when FBS was used to prepare MSCs, demonstrating reduced efficacy of FBS prepared MSCs. Fetal bovine serum should no longer be utilized for MSC preparation in pre-clinical study, clinical study, or veterinary applications. The use of FBS in previously reported studies, and in MSC therapeutics with current foreign market authorization, should be considered when interpreting results.

## Introduction

Despite decades of work, consistent and reproducible clinical efficacy of mesenchymal stem cells (MSCs) has not been demonstrated (1–3). Failure to meet clinical endpoints in both late phase clinical trials and post-approval monitoring has precluded market authorization in the United States (1–4). Likewise, lack of predictable efficacy of non-human MSCs plagues the veterinary community and has casted doubt on the usefulness of MSCs, both translationally and clinically. One reason for the lack of consistent efficacy may be MSC preparation technique (1). Pittenger et al. recently emphasized the importance of MSC preparation technique, stating that the preparation method of MSCs is the product (5). Preparation methods include culture media composition and serum supplement sources.

Supplementation of culture media with fetal bovine serum (FBS) has been a standard MSC preparation technique since MSCs were first described in the 1970s, providing growth factors, hormones, and other undefined, yet essential, components to cell culture media (6). However, the use of FBS is decreasing because of ethical concerns, availability, and the risk of disease transmission from bovine products (7). Despite this shift in FBS acceptance, FBS supplemented MSCs have market approval for use in humans in Canada and New Zealand, and FBS supplementation remains the industry standard in pre-clinical and veterinary MSC use (4, 5, 8–11).

An important, but infrequently discussed, consequence of FBS supplementation during MSC preparation is the accumulation of intracellular bovine contamination that is presented on MHCI, which leads to seroconversion of the recipient (12–15). In horses, we confirmed that the accumulation of intracellular bovine proteins by MSCs leads to local inflammation after therapeutic administration, but did not assess anti-bovine titers (16). In that report, removal of FBS during the final 48 h of culture markedly reduced intracellular bovine contamination, but all MSCs remained positive for intracellular bovine proteins (16).

A lack of change in anti-bovine titers in horses and cats after MSC therapy has led others to conclude that FBS contamination is not clinically relevant yet, in humans there is evidence that seroconversion against bovine proteins in MSC recipients correlates to poor clinical response (11, 17, 18). The question remains, what do pre- and post-MSC treatment anti-bovine titers mean in patients receiving FBS supplemented MSCs? Immune recognition of intracellular bovine proteins and resultant cytotoxicity could explain why pre-clinical study often fails to predict therapeutic response and why human clinical trials have failed to meet rigorous clinical endpoints in the United States (2, 5, 9, 19).

Our objective was to determine if there is an immune response against autologous MSCs because of laboratory preparation with FBS. First, we confirmed that replacement of FBS supplementation with bone marrow supernatant (BMS) supplementation did not alter MSC growth or characterization. In the equine model, we then performed repeated intra-articular injections of autologous FBS supplemented MSCs (FBS-MSCs) or autologous BMS supplemented MSCs (BMS-MSCs). We demonstrate immune recognition with antibody mediated death of MSCs, local inflammation, and reduced efficacy after FBS-MSC administration, which did not occur with BMS-MSCs. Given the historical and ongoing use of FBS in pre-clinical and clinical trials, identifying FBS use and potential recipient immune recognition with subsequent antibody mediated MSC death is imperative in interpreting results. In future study, especially pre-clinical study and veterinary applications where FBS supplementation remains the standard practice, FBS should not be utilized.

## Materials and Methods

### Animals and Experimental Overview

All animals were cared for according to university standards and all procedures were approved by the animal care and use committee (AUP 2018-0003 and 2018-0118). Six horses were utilized for *in vitro* experiments (BMS characterization; five females, one castrated male), and 18 horses were utilized for the *in vivo* experiments (fetlock model; 13 females, five castrated males). All horses were Quarter Horse type and ranged from 4 to 22 years of age (median, 13; Table 1). For the *in vivo* experiment, the left metacarpophalangeal joint was utilized, and all joints were healthy with no known joint pathology. Horses were randomly assigned to groups by drawing group assignment from a hat (FBS, autologous BMS, pooled BMS).

**Table 1:** Age, and gender (F = female; G = gelding, castrated male) of horses in both the *in vitro* and *in vivo* experiments.

	**Group Assignment**	**Age**	**Gender**
***In vitro***			
Horse 1	*In vitro*	14	F
Horse 2	*In vitro*	16	F
Horse 3	*In vitro*	8	F
Horse 4	*In vitro*	12	F
Horse 5	*In vitro*	9	F
Horse 6	*In vitro*	4	F
***In vivo***			
Horse 1	FBS	18	F
Horse 2	Autologous BMS	16	F
Horse 3	Autologous BMS	12	F
Horse 4	Pooled BMS	12	G
Horse 5	FBS	22	G
Horse 6	Autologous BMS	4	G
Horse 7	Pooled BMS	13	F
Horse 8	Pooled BMS	14	F
Horse 9	FBS	12	F
Horse 10	Pooled BMS	11	F
Horse 11	Autologous BMS	12	G
Horse 12	Autologous BMS	12	F
Horse 13	FBS	13	F
Horse 14	Pooled BMS	13	F
Horse 15	FBS	12	F
Horse 16	Pooled BMS	11	F
Horse 17	Autologous BMS	19	G
Horse 18	FBS	18	F

*All MSCs were autologous*.

Bone marrow obtained from six horses was portioned to two groups for MSC isolation and expansion in media supplemented with autologous BMS (one group, *n* = 6) or FBS (second group, *n* = 6). *In vitro* growth rate, characterization, and immunomodulation were compared in a paired analysis.

For *in vivo* evaluation, MSCs were isolated and expanded in autologous (*n* = 6) or pooled (*n* = 6) BMS, or FBS (*n* = 6) and cryopreserved in recipient serum. Intra-articular injection of autologous FBS-prepared MSCs (*n* = 6) or autologous BMS-prepared MSCs (pooled BMS, *n* = 6; or autologous BMS, *n* = 6) was performed on days 0 and 29. When no differences in clinical assessments were detected between groups when MSCs were prepared with autologous or pooled BMS, data were combined (BMS and pooled) for FBS (*n* = 6) vs. BMS (*n* = 12) testing. To mimic naturally occurring inflammation, aqueous lipopolysaccharide (LPS) was injected immediately prior to MSCs at the second intra-articular injection. Clinical reaction, synovial cytology, synovial cytokines, synovial MSC concentration and cytotoxicity against MSCs were evaluated for the week after each intra-articular injection.

### MSC Isolation and Expansion

Bone marrow was collected from the sternum as previously described (20). For the *in vitro* portion, ~120 mls of bone marrow was collected. For the *in vivo* portion, ~360 mls of bone marrow was collected. Heparinized bone marrow was centrifuged at 300 g for 5 min and the BMS was collected and filtered. The cellular fraction underwent red blood cell lysis as previously described (21). Briefly, red blood cell lysing solution (7.7 mg/ml NH4CL; 2.06 mg/ml hydroxymethane–aminomethane, pH 7.2) was added to the cellular fraction and centrifuged twice (300 g for 5 min). The cells were washed in DPBS (Dulbecco's phosphate buffered saline, Corning) and re-suspended in serum free media [Dulbecco's modified Eagle's medium (DMEM, Corning), 1 g/l glucose supplemented with 10,000 U/ml Penicillin; 10 mg streptomycin sulfate, 25 μg/ml amphotericin B (Gibco); 2.5% HEPES buffer (Life Technologies); 10 μg/ml human recombinant basic fibroblastic growth factor (b-FGF, Corning)]. Media contained either 10% BMS (autologous or pooled) or 10% FBS (HyClone) and MSCs were maintained at 37°C in 5% CO_2_, humidified air with media that was replaced with fresh media 3 times per week. Pooled BMS was created with equal parts from each of the six horses in the autologous BMS group.

After 7 days in culture, MSCs were passaged and re-plated at 5,000–7,000 MSCs/cm^2^, which was repeated each time confluence reached 70–80% until the third passage. Once MSCs reach the third passage (P3), they were cryopreserved is cryopreservation media (95% autologous serum and 5% DMSO) as previously described (21).

### Characterization of BMS-MSCs and FBS-MSCs

#### In Vitro Colony Forming Unit-Fibroblast Assay

The equivalent of 1 ml of raw bone marrow was plated to 10 cm tissue culture dishes and maintained in media supplemented with 10% BMS or 10% FBS. After 10 days, colonies were stained with 3% crystal violet (Sigma Aldrich) and counted.

#### Population Doubling Time

Population doubling time was calculated using the following equation: PDT = days in culture ^*^ log 2/(log*f* –log*i*) where *f* is final cell count and *i* is the initial number of cells.

#### Trilineage Differentiation and Cell Surface Marker Expression

MSCs at passage 3 underwent trilineage differentiation into adipocytes, chondrocytes, and osteocytes, and cell surface marker expression of MHCI, MHCII, CD29, CD45, and CD90 was evaluated as previously described (21, 22).

#### Mixed Lymphocyte Reactions

Previously cryopreserved MSCs were thawed and plated at 50,000 cells per well for 24 h prior to inactivation with mitomycin C (Sigma Aldrich) as previously described (23). Responder and stimulator lymphocytes were isolated from two unrelated donors using a Ficoll (GE Healthcare) gradient with the addition of carbonyl iron (Sigma Aldrich) (24). Stimulator lymphocytes were inactivated by incubation with 50 μg/ml mitomycin C for 30 min and then added at a density of 1 x 10^6^ stimulator lymphocytes per well. Responder lymphocytes were stained with a commercially available nuclear stain (CellTrace® Violet, Thermo Fisher) and 2 x 10^6^ responder lymphocytes were added to each well. Cultures were maintained for 5 days, after which lymphocytes were collected and stained with anti-equine CD3+ antibody (UC Davis) at a 1:200 dilution. Flow cytometry was then performed on CD3+ T lymphocytes to assess proliferation with the use of commercially available software (FlowJo™ Software). Stained, unstimulated responder lymphocytes were used as a negative proliferation control, Concanavalin A (Sigma Aldrich) stimulated responder lymphocytes were used as a positive proliferation control, and changes in mean fluorescence intensity were evaluated as a percent change from the negative control as previously described (25).

### Equine Model

On day 0, 10 x 10^6^ autologous MSCs prepared respective of group assignment were thawed at 37°C and administered to the left metacarpophalangel joint by intra-articular injection in cryopreservation media (95% autologous serum and 5% DMSO). On day 29, autologous MSC injection was repeated with 25 ng LPS (lipopolysaccharide from Escherichia coli O55:B5, Sigma Aldrich) suspended in saline and injected immediately prior to MSCs.

#### Clinical Evaluation

Physical examinations including heart rate, respiratory rate, and temperature were performed prior to and every 12 h for 3 days after each injection. As a measurement of pain, gait asymmetry was quantified using an inertial-based sensor system (Lameness Locator, Equinosis®) prior to and after each injection (days 0, 1, 2, 3, 7, 29, 30, 31, 32, and 36). Peri-articular edema and synovial effusion were scored at the same time points; 0 = no edema/effusion, 1 = mild edema/effusion, 2 = moderate edema/effusion, 3 = severe edema/effusion. Limb circumference was measured at the level of distal metacarpophalangeal IV on days 29, 30, 31, 32, and 36.

#### Synovial Cytology, Cytokine and Chemokine Analysis

Synovial fluid was collected from the left metacarpophalangeal joint prior to and after each injection on days 0, 1, 2, 3, 7, 29, 30, 31, 32, 36 and examined by a board-certified veterinary pathologist. Any effect of repeated synoviocentesis would have equal chance to affect both groups (26, 27). Synovial fluid analysis including total nucleated cell count, cellular differential, and total protein measurement was performed on all samples.

Synovial fluid collected on days 1 and 30 was also analyzed using a 23 analyte, equine specific, multiplex kit (Millipore Sigma) as previously described (28). Analytes measured included: FGF-2, eotaxin, G-CSF, IL-1α, GM-CSF, fractalkine, IL-13, IL-5, IL-18, IL-1β, IL-6, IL-17a, IL-2, IL-4, IL-12, IFNγ, IL-8, IP-10, GRO, MCP-1, IL-10, TNFα, and RANTES.

#### Synovial Colony Forming Unit-Fibroblast Assay

On days 1, 7, 30, and 36, eight drops of synovial fluid from the 20 g 1.5 inch needle, or ~1 ml, were plated to a 10 cm tissue culture dish along with MSC culture media. Media was changed after 24 h and again 72 h later. After 7 days in culture, dishes were stained with 3% crystal violet, allowed to dry overnight, and colonies counted without magnification.

#### Anti-FBS Antibody Concentrations

Blood was collected on all horses weekly prior to injection and for 8 weeks after the first injection (days 0, 7, 14, 21, 28, 35, 42, 49, and 56). An anti-FBS antibody ELISA was performed as previously described (29, 30). Briefly, plates were coated with FBS from the same lot as MSC preparation, and incubated overnight. Plates were washed, serum was added at a 1:3,200 dilution, and for 30 min. Secondary antibody (Abcam) was added at 1:20,000 dilution for 30 min. After a final wash, 100 μl of TMB (Genway Biotech Inc.) was added followed by 100 μl of stop solution (Genway Biotech Inc.) 15 min later. Plates were read at 450 nm, and optical density (OD) reported. Fetal equine serum (FES) was used as a negative assay control, which had the same optical density as the blank control. Titers were then measured by repeating the above ELISA procedure with serial dilutions (from 1:1,600 to 1:819,200) of serum collected from the FBS group on days 0 and 56.

#### Microcytotoxicity Assay

Microcytotoxicity assays were performed using serum collected weekly (days 0, 7, 14, 21, 28, 35, 42, 49, and 56) and synovial fluid collected prior to and after the first and second injections (days 0, 1, 7, 30, and 36) with autologous MSCs cultured in either BMS or FBS. Briefly, 2 μl of serum or synovial fluid was added to a Terasaki plate and 5 μl of paraffin oil (Sigma Aldrich) layered on top. Autologous MSCs were suspended in DPBS at a concentration of 1,000 cells/μl and 2 μl of the suspension added to each well, ensuring that the cell solution was in contact with the serum or synovial fluid. After 30 min at room temperature, 5 μl of rabbit complement was added (One Lambda) and plates were incubated for another 60 min at room temperature. Two μl of 5% eosin (Sigma Aldrich) was then added to each well, after 5 min 5 μl of 10% formalin (Thermo Scientific) was added. Cells were allowed to settle overnight and percentage of cell death was visually assessed at 20X magnification within 24 h, in a blinded manner as previously described (31). Fetal equine serum was used as a negative control and MHCI specific monoclonal antibody (CZ3.2, provided by Donald Miller), was used as a positive control.

#### Immunoglobulin Depletion

To confirm that cell death in the microcytotoxicity assays was anti-FBS antibody mediated, immunoglobulins were depleted from serum collected from FBS-MSC recipients on day 35 as previously described (32). Briefly, a commercially available kit with a Protein A column (ProteoExtract®, Merck KGaA) was used followed by manual depletion with Sepharose G beads (Millipore Sigma). One hundred μl of serum was diluted in 900 μl of 1x binding buffer. Samples were passed through the Protein A column to remove IgG, in a dropwise manner resulting in partial IgG removal. Two hundred μl of preconditioned Sepharose G beads was added to 300 μl of undiluted eluate and incubated for 1 h with gentle mixing for complete IgG removal. Microcytotoxicity assays were repeated with undiluted serum, serum diluted in 1x binding buffer, partial immunoglobulin depleted serum, or complete immunoglobulin depleted serum. Assays were completed in duplicate, with donor MSCs cultured in either BMS or FBS.

#### Statistical Analysis

Differences in *in vitro* data between groups were evaluated by paired Wilcoxon signed rank. *In vivo*, edema, effusion, limb circumference, and lameness were normalized to baseline (day 0 and day 29) prior to MSC injection. Differences between groups and over time in edema, effusion, limb circumference, lameness, and number of colonies present were tested using Kruskal Wallis or Wilcoxon rank sum tests. As a follow-up, a mixed model was used for all *in vivo* data, no differences were found between either analysis. Differences in proportion of joints positive for MSC colonies were tested using Fisher's exact test at each time point. Groups were considered different when the *p* < 0.05.

## Results

### Greater MSC Isolation With BMS, but no Difference in Expansion, Characterization, or Immunomodulatory Capacity in BMS-MSCs Compared to FBS-MSCs

**Figure 1:**
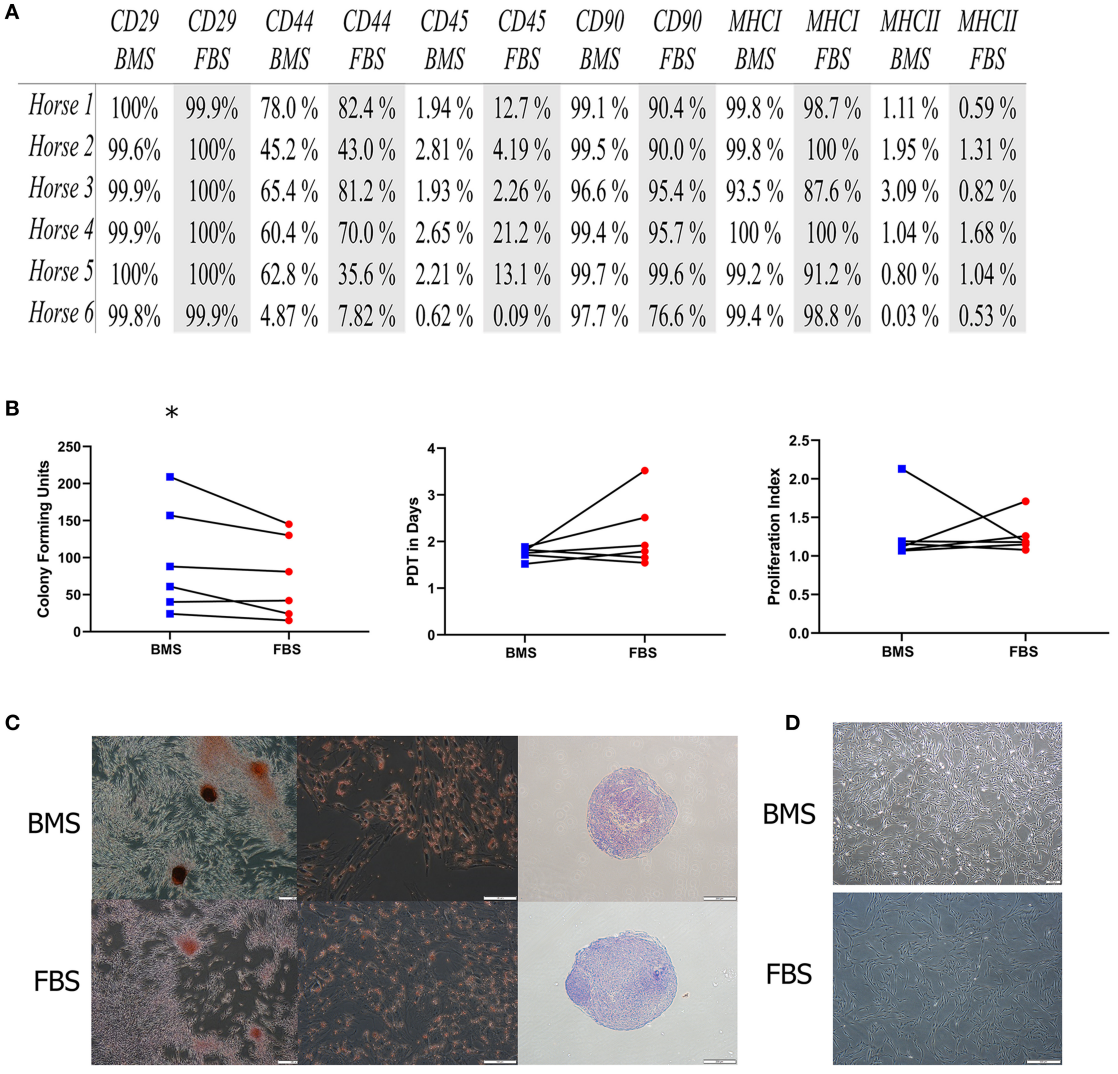
MSC isolation is increased in BMS-MSCs compared to FBS-MSCs; expansion and characterization are not different. **(A)** Table of percentage of cell surface marker present on MSCs; there was no difference in cell surface marker expression. **(B)** There was greater isolation of CFU-f colonies with BMS compared to FBS (^*^p ≤ 0.05), but no difference in population doubling time (PDT) from isolation to passage 3, or immunomodulation, measured by lymphocyte proliferation index in mixed lymphocyte reactions. **(C)** Trilineage differentiation into bone, fat, and cartilage was not different between BMS-MSCs and FBS-MSCs. **(D)** BMS-MSCs were similar in appearance to FBS-MSCs.

First, we investigated whether BMS supports MSC isolation and expansion without differences to MSC characterization or immunomodulatory capacity compared to FBS supplementation. There was an increased rate of MSC colony isolation after BMS supplementation compared to FBS, but no differences in expansion rate between BMS-MSCs and FBS-MSCs (Figure 1).

No differences in immunomodulatory function were seen using modified one-way mixed lyphocyte reactions (Figure 1). After three passages, BMS-MSCs and FBS-MSCs were phenotypically similar without appreciable differences in morphology, and there were no differences in cell surface marker expression or trilineage differentiation into bone, cartilage, and fat (Figure 1).

### FBS-MSCs, but Not BMS-MSCs, Cause Local Inflammation and Are Targeted by the Recipient Immune System

Eighteen horses received intra-articular injection of autologous MSCs prepared with media supplemented with autologous BMS (*n* = 6), pooled BMS (*n* = 6), or FBS (*n* = 6). Intra-articular injections occurred on experimental day 0 and 29. There were no differences between the autologous and pooled BMS-prepared groups in clinical or laboratory findings including lameness, synovial CFU-f, edema, effusion, cytokine and chemokine concentrations, or cytotoxicity of MSCs; therefore, pooled and autologous BMS data were combined to a single group (BMS-MSC, *n* = 12) and compared to FBS (*n* = 6).

#### FBS Contamination Causes Local Inflammation and Adverse Clinical Response

**Figure 2:**
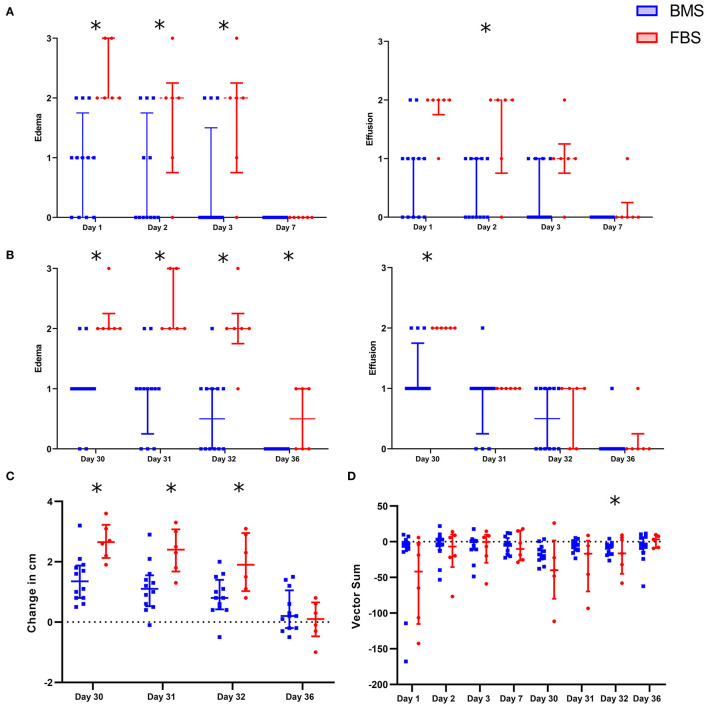
Local adverse response in FBS-MSC recipients compared to BMS-MSC recipients. **(A)** Peri-articular edema and synovial effusion was worsened in FBS-MSC recipients (significance denoted by asterisks, ^*^p ≤ 0.05). **(B)** After the second injection, with concurrent administration of LPS, peri-articular edema and synovial effusion was again, worsened in FBS-MSC recipients. **(C)** Likewise, limb circumference was increased after the second injection in FBS-MSC recipients. **(D)** There was no difference in gait asymmetry (pain) after the first injection. After the second injection, with concurrent LPS administration, there was a trend of worsened gait asymmetry in FBS-MSC recipients on day 30 and 31, and worsened gait asymmetry on day 32 in FBS-MSC recipients.

**Figure 3:**
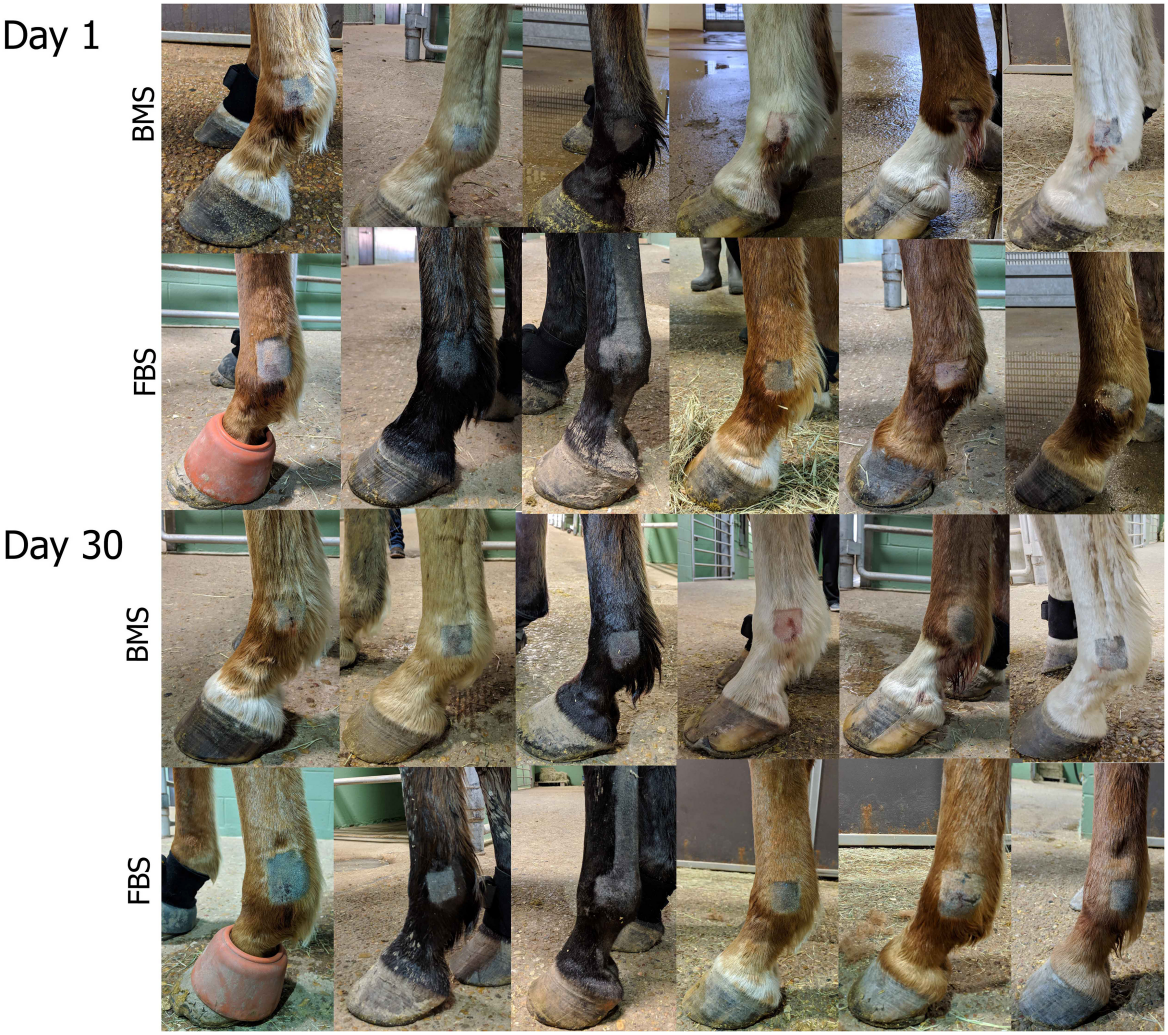
Post-injection edema was marked in FBS-MSC recipients compared to BMS-MSC recipients. Photographs taken on days 1 and 30, one day after FBS-MSC or BMS-MSC injection. There was worse swelling that retained impression (i.e., pitting edema) in FBS-MSC recipients.

After each intra-articular injection, there was increased peri-articular edema and synovial effusion in FBS-MSC recipients compared to BMS-MSC recipients (Figures 2, 3) (33, 34). There were no differences in pain between groups after the first injection. One FBS-MSC recipient was removed from gait analysis for assessment of pain after the second injection because of a right forelimb lameness, not related to the study. After the second MSC injection, when LPS was also administered, there was reduced MSC efficacy with a trend of more pain in FBS-MSC recipients on day 30 and 31, and significantly worse pain in FBS-MSC recipients on day 32 (Figure 2) (9, 18).

#### No Difference in Synovial Fluid Cytology, Cytokine or Chemokine Concentrations

**Figure 4:**
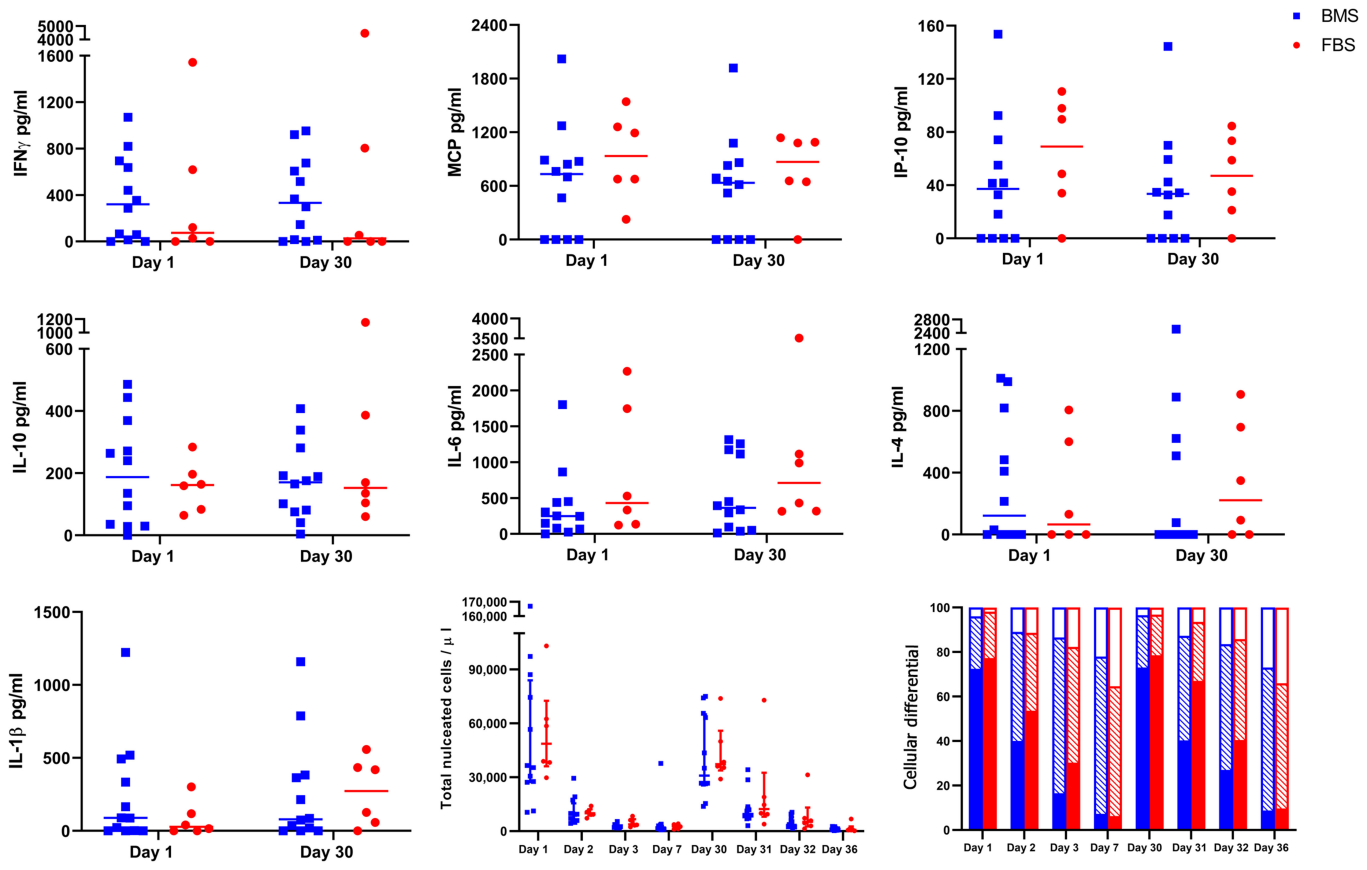
No differences in cytokine and chemokine concentration, total nucleated cell count, or cellular differential after either injection on days 0 and 29. There were no differences in concentration of IFNγ, MCP, IP-10, IL-10, IL-6, IL-4, IL-1β in synovial fluid on days 1 or 30 between groups. Likewise, there were no differences in synovial total nucleated cell count or cellular differential after either injection (solid portion = neutrophils; diagonal lines = small lymphocytes; empty portion = large mononuclear cells).

There were no differences in synovial fluid cytology (total nucleated cell count or proportion of cell type) after either injection (Figure 4). Synovial cytokines and chemokines revealed measurable concentrations of IFNγ, MCP, IP-10, IL-10, IL-6, IL-4, and IL-1β, but no differences between groups the day after each injection (Figure 4). This single time point may have missed differences due to LPS, but were the same time points as our recent report of significant synovial differences due to allogeneic MHCI mismatch (28).

#### Anti-bovine Antibodies Were Present and Unchanged in all Horses

**Figure 5:**
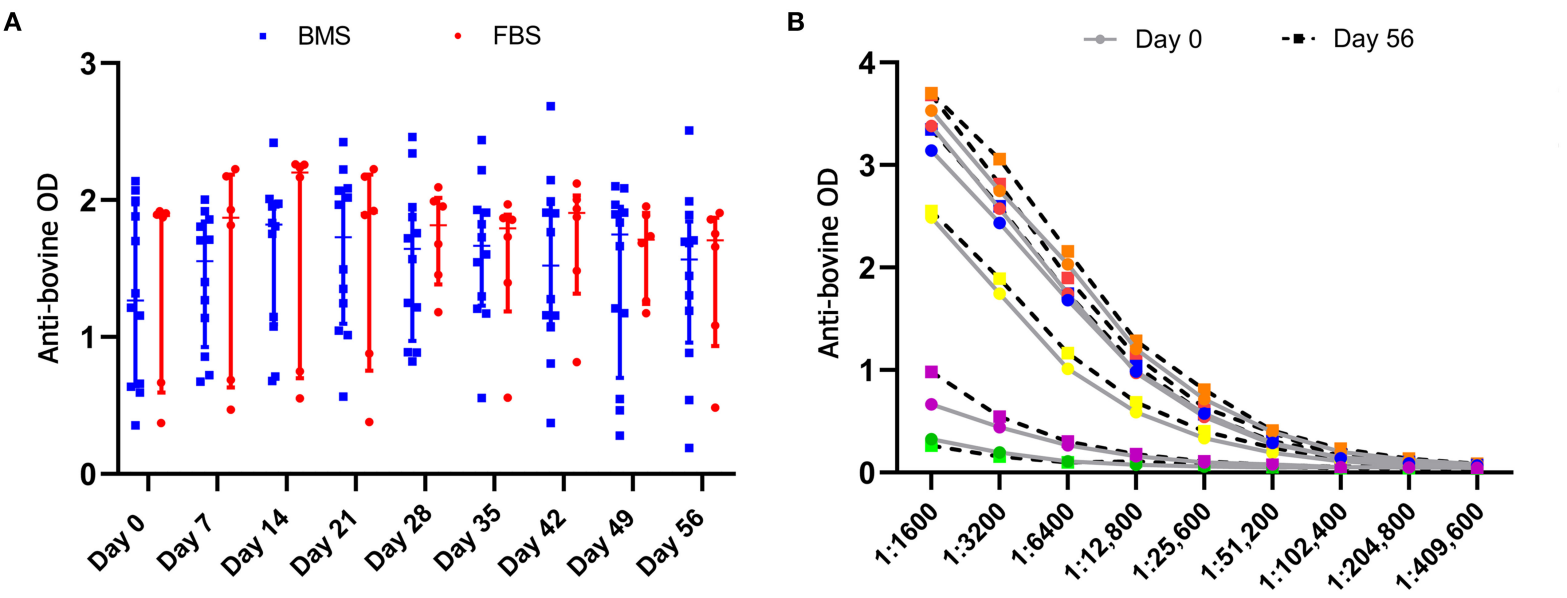
Anti-bovine titers were present in all horses prior to MSC administration that did not change after MSC administration. **(A)** There were no differences in antibody concentrations, measured by optical density (OD), between groups, and antibody concentrations did not change over time. **(B)** There was no difference in anti-bovine titers in FBS-MSC recipients prior to and after FBS-MSC exposure. Each individual is represented by a different color.

Anti-bovine antibody concentrations were not different between groups or over time (Figure 5). Titers varied by individual, with a median maximum titer of 1:204,800 (range, 1:12,800–1:409,600) without differences in anti-bovine antibody titers between day 0 and 56 (Figure 5).

#### Anti-bovine Antibodies in the Recipient Cause Death of FBS-MSCs, but Not BMS-MSCs

**Figure 6:**
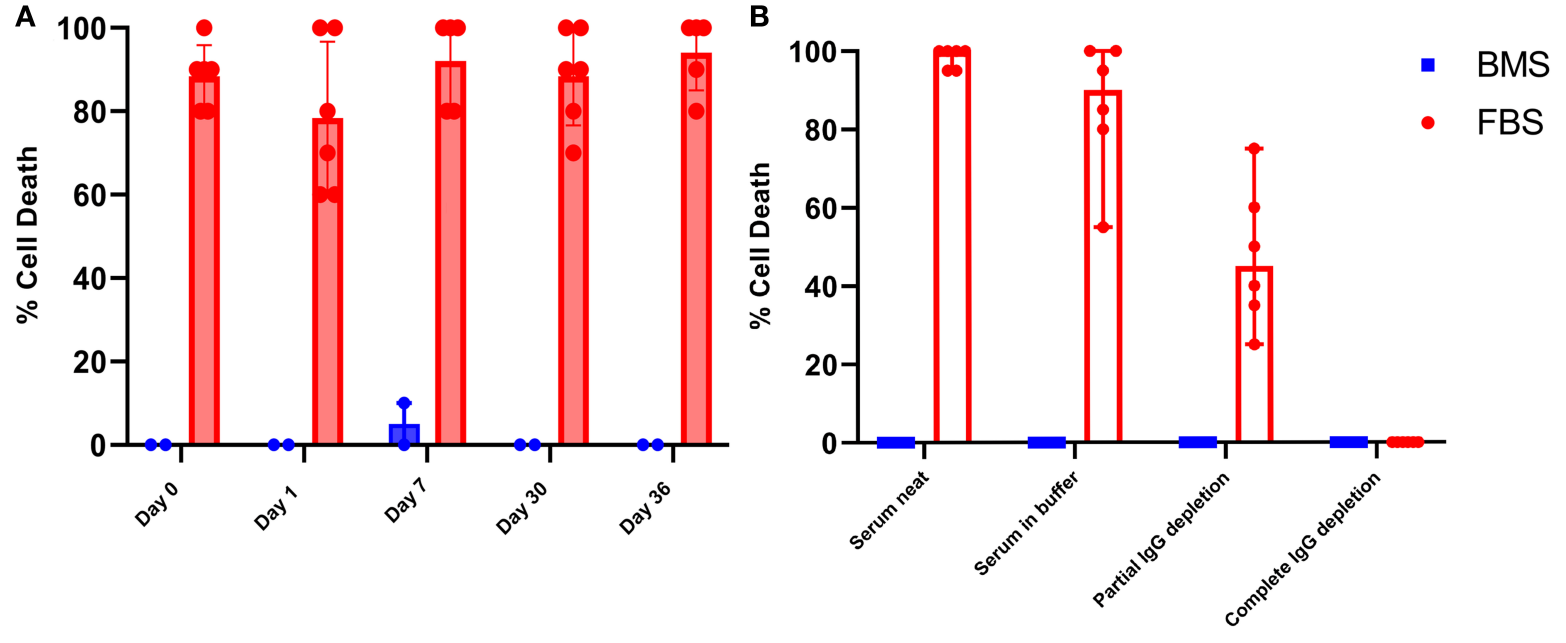
Antibodies against bovine proteins are present in serum and synovial fluid, and cause death of FBS prepared MSCs. **(A)** Synovial fluid collected on days 0, 1, 7, 30, and 36 produced significant cell death when combined with autologous FBS-MSCs. **(B)** To demonstrate that MSC cell death was antibody mediated, serum collected on day 35 was depleted of immunoglobulins partially and completely. All serum samples were combined with either BMS-MSCs or FBS-MSCs. There was no death of BMS-MSCs, there was significant cell death of FBS-MSCs both neat and in buffer, a reduction of cell death with partial IgG depletion, and elimination of cell death with complete IgG depletion.

While it is well documented that anti-bovine antibodies are present in human and equine serum, little is known about the consequence of these antibodies relative to FBS-MSCs (35, 36). It has been considered that the immune modulating properties of MSCs could prevent recipient immune recognition of bovine proteins from FBS that are presented by MHCI as part of normal cellular surveillance (37). Microcytotoxicity assays with recipient serum and autologous MSCs resulted in widespread death of FBS prepared MSCs. In contrast, there was virtually no cytotoxicity of BMS-MSCs (Figure 6).

To confirm that death of FBS-MSCs in the microcytotoxicity assay was due to antibody, we repeated mircrocytoxocity assays with partially and fully immunoglobulin depleted serum. There was virtually no cell death of BMS-MSCs when combined with serum in buffer, partial, or complete immunoglobulin depleted serum. In contrast, there was marked death of FBS-MSCs when combined with serum in buffer, a reduction in cell death with partial immunoglobulin depletion, and absence of cell death after complete immunoglobulin depletion (Figure 6).

#### Pre-existing Anti-bovine Antibodies in Synovial Fluid Cause FBS-MSC Death

After demonstrating the presence of consistent and unchanged anti-bovine antibodies in serum capable of causing death of FBS-prepared MSCs, we wanted to evaluate if antibodies are of consequence in the articular environment. This is of particular importance because the articular environment is often considered to be immune privileged, as it is nearly acellular with a distinct blood-joint barrier that minimizes diffusion of small molecules (38, 39). We repeated microcytotoxicity assays combining FBS-MSCs or BMS-MSCs with synovial fluid collected on days 0, 1, 7, 30, and 36 after intra-articular MSC administration. At all time-points, there was cytotoxic FBS-MSC death but not BMS-MSC, confirming that anti-FBS antibodies are present in synovial fluid in sufficient quantities to cause cytotoxic cell death of FBS contaminated MSCs before and after intra-articular injections (Figure 6).

#### Joints Injected With FBS-MSCs Have Lower Synovial MSC Concentrations Compared to Those Injected With BMS-MSCs

**Figure 7:**
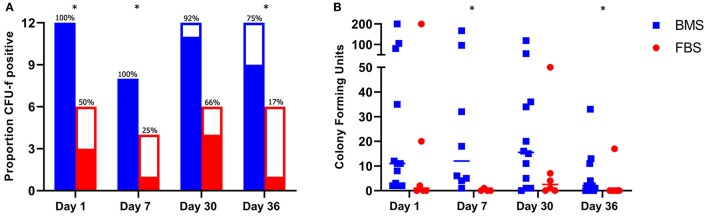
Increased synovial MSC isolation after BMS-MSC administration compared to FBS-MSC administration. **(A)** The proportion of synovial fluid CFU-f plates with at least one colony (filled section of the bar) was higher in BMS-MSC recipients [significance denoted by asterisks, ^*^p ≤ 0.05]. Bars represent total number of CFU-f cultures performed per day with solid portions denoting when CFU-f colonies were present and clear portion denoting a lack of CFU-f colonies. **(B)** The synovial MSC concentration was higher in BMS-MSC recipients 1 week after each injection.

To assess for differences in MSC survival within the joint, we measured synovial MSC concentrations using CFU-f assays on days 1, 7, 30, and 36; the measured MSCs may have been either endogenous progenitors within synovial fluid or injected MSCs that had survived. The proportion of CFU-f plates with at least one colony was higher on days 1, 7, and 36 in BMS-MSC recipients compared to FBS-MSC recipients (Figure 7). The total number of colonies from each joint was higher 1 week after each injection on days 7 and 36 in BMS-MSC recipients compared to FBS-MSC recipients (Figure 7).

## Discussion

We demonstrate that the recipient immune response to FBS prepared autologous MSCs results in MSC death, local inflammation, and reduced synovial MSC concentrations. Recent failures of therapeutic MSCs to achieve market approval in the United States, despite foreign regulatory approval, might be due to FBS use during MSC preparation resulting in altered clinical effect and failure to meet stringent end points (4, 8, 9, 35, 40). While clinical human MSC preparation is transitioning away from FBS use, continued FBS supplementation of MSCs in animal models and veterinary clinical application will obfuscate clinical translation (9, 34, 41).

Our findings refute the conclusion that anti-bovine titers are not of consequence because they do not change with repeated exposure to FBS-MSCs (11, 14, 17). We suggest the lack of change in anti-bovine titers, described by others and here, is because peak titers that are incapable of an anamnestic response exist prior to MSC therapy (11, 17). In horses, these peak titers are due to routine bi-annual vaccination against viral pathogens, which are also prepared with FBS (36). This frequent vaccination results in inadvertent, but thorough, vaccine induced immunity and peak titers against bovine proteins (36).

Veterinary reports have attributed inflammatory events after MSC therapy to a normal physiologic response to MSCs, and in people immunomodulatory drugs are commonly administered to mitigate inflammation during MSC administration (9, 17, 34, 42–44). We show that adverse reactions and local inflammation are not the normal physiologic response to MSCs, rather a consequence of FBS contamination of MSCs (33, 34). Moreover, the articular environment that we used is particularly well suited to investigate these adverse events because the blood-joint barrier and large volume-to-surface area ratio sequesters the local response, augmenting detection of inflammation (38, 39). Given this exquisite sensitivity of the articular joint to inflammation, the absence of edema or effusion in BMS-MSC recipients without concurrent anti-inflammatory administration or limb wrapping support is remarkable.

In a recent report from our group using the same experimental model, we showed differences in synovial cytokine concentrations, including IFNγ, 1 day after mismatched allogeneic MSCs were administered compared to matched allogeneic MSCs (28). Intriguingly, in the current report, we did not see differences in synovial cytokines at this same time point, despite marked synovial effusion and peri-articular edema that lasted several days in FBS-MSC recipients. This difference in degree and duration of inflammation, with greater degree of inflammation in mismatched recipients (higher IFN-gamma on days 1 and 30) reported previously and longer duration of inflammation in FBS-MSC recipients (more peri-articular edema for the entire week after each intra-articular injection) reported here, is likely because of differences in the mechanism of antigen recognition. With allogeneic mismatch, MHCI incompatibility would result in every MSC being immediately identified as foreign. Whereas, FBS prepared MSCs presenting cellular contents in routine cell monitoring may not initially be presenting bovine antigen on their MHCI, may be presenting very little bovine antigen on their MHCI, or may not present bovine antigen on their MHCI until days after administration. It is also possible that the prolonged edema and effusion in the FBS-MSC group is due to exocytosis of bovine antigen by FBS prepared MSCs or by recognition of bovine antigen by IgE, which is often present in horses (36).

We also recently reported increased endogenous progenitors in joints injected with MHCI matched allogeneic MSCs compared to mismatched allogeneic MSCs (28). In the report here, all injected MSCs were autologous and whether the increased synovial MSC concentration after BMS-MSC administration was due to improved MSC persistence or endogenous progenitor upregulation cannot be distinguished. Nonetheless, recipient immune targeting of FBS-MSCs resulted in lower synovial MSC concentrations.

Continued acceptance of FBS for veterinary and pre-clinical study may be due to the marked failures of platelet products to adequately support FBS-free preparation of animal derived MSCs, the high cost of chemically define media, and the well-known failure of adult derived serum to support MSC isolation and expansion (45–48). After years of failure by our group to develop equine platelet lysate or releasate for isolation and long-term expansion without alteration of MSC characteristics (data not shown), we demonstrated that replacement of FBS with autologous serum for 48 h greatly reduces FBS contamination of equine MSCs, but does not eliminate it (16). In our efforts to eliminate FBS contamination, we discovered BMS as an FBS alternative. Advantages of BMS is that it can be autologous, is inexpensive to obtain and process, and is a by-product when utilizing bone marrow derived MSCs. A disadvantage is that large volumes of bone marrow are required to support isolation and expansion for the entire MSC culture duration.

Given the unchanged growth and metabolism of BMS-MSCs as compared to FBS-MSCs, it is possible that BMS in the final stages of MSC preparation could be used to more thoroughly eliminate FBS contamination as compared to adult derived serum (16). Additionally, we do not know which factor, or factors, is present in BMS and not in adult derived serum. In the absence of this knowledge, we assessed for BMS quality differences by comparing to FBS *in vitro* and by comparing autologous and pooled BMS *in vivo*, which did not have differences. This suggests that BMS quality was consistent in this group of horses, but further work is needed.

We show that recipient anti-bovine titers cause antibody mediated death of MSCs that have been prepared with FBS, with resultant local inflammation and reduced synovial MSCs after intra-articular administration. The historic and current use of FBS for MSC preparation is likely to misrepresent MSC effect because of cytotoxicity and adverse responses to MSCs with FBS contamination (10, 42, 49). When evaluating reported pre-clinical, veterinary, and human clinical trials, the use of FBS should be considered when interpreting results (4, 9, 40, 41). Bone marrow supernatant is a simple, inexpensive, and autologous replacement for FBS that eliminates immune targeting and the resultant adverse clinical effects. Fetal bovine serum should not be used for MSC supplementation in veterinary, pre-clinical or clinical MSC preparation, and the use of BMS should be further investigated.

## Data Availability Statement

The raw data supporting the conclusions of this article will be made available by the authors, without undue reservation.

## Ethics Statement

The animal study was reviewed and approved by Texas A&M University Institutional Animal Care and Use Committee.

## Author Contributions

AR and GL: study design, data acquisition, analysis and interpretation of data, and manuscript preparation. MB: data acquisition, analysis and interpretation of data, and manuscript preparation. AW: study design, analysis, data acquisition and interpretation of data, and manuscript preparation. All authors contributed to the article and approved the submitted version.

## Funding

Funding was provided by the Link Endowment for Equine Research at Texas A&M University, and the Linda and Dennis H. Clark '68 Endowed Chair for Equine Studies.

## Conflict of Interest

The authors declare that the research was conducted in the absence of any commercial or financial relationships that could be construed as a potential conflict of interest.

## Publisher's Note

All claims expressed in this article are solely those of the authors and do not necessarily represent those of their affiliated organizations, or those of the publisher, the editors and the reviewers. Any product that may be evaluated in this article, or claim that may be made by its manufacturer, is not guaranteed or endorsed by the publisher.
